# Relationship between Urinary Calcium Excretion and Lower Urinary Tract Symptoms

**DOI:** 10.3390/metabo12030229

**Published:** 2022-03-05

**Authors:** Tomohiro Matsuo, Hidenori Ito, Kensuke Mitsunari, Kojiro Ohba, Yasuyoshi Miyata

**Affiliations:** Department of Urology, Nagasaki University Graduate School of Biomedical Sciences, Nagasaki 852-8501, Japan; ito218@nagasaki-u.ac.jp (H.I.); kmitsunari@nagasaki-u.ac.jp (K.M.); ohba-k@nagasaki-u.ac.jp (K.O.); yasu-myt@nagasaki-u.ac.jp (Y.M.)

**Keywords:** urinary calcium, overactive bladder, nocturia

## Abstract

To date, few detailed studies have been conducted on the convenient and useful markers for the prevalence of lower urinary tract symptoms (LUTS), including overactive bladder (OAB) and nocturia. A high level of calcium (Ca) excretion (hypercalciuria) is indicative of lifestyle-related diseases such as hypertension, which are associated with the onset of LUTS. Hence, in this study we attempted to clarify the relationship between urinary Ca excretion and OAB, nocturia, and nocturnal polyuria in adults. The present study showed that patients with hypercalciuria frequently experienced OAB, nocturia, and nocturnal polyuria. In addition, this study revealed that the severity of LUTS is significantly associated with urinary Ca excretion and that hypercalciuria is an important risk factor for OAB, nocturia, and nocturnal polyuria.

## 1. Introduction

In the field of pediatric urology, excessive excretion of calcium (Ca) from the kidneys into urine induces nocturnal polyuria and decreases functional bladder capacity, which is one of the causes of nocturnal enuresis and other lower urinary tract symptoms (LUTS) [1,2,3]. Even adult patients with typical lifestyle-related diseases, such as hypertension and osteoporosis, generally have a large amount of urinary Ca excretion (hypercalciuria), which can cause LUTS such as overactive bladder (OAB) and nocturia [4]. A representative pathogenic mechanism of hypercalciuria in hypertensive patients has been recognized that is, the increase in urinary calcium excretion has been associated with urinary excretion of excessive sodium, which is one of the most important components of hypertension [5]. In addition, in hypertensive patients, parathyroid hormone is secreted to control urinary Ca excretion, but chronic stimulation of the parathyroid gland accelerates bone turnover, promotes osteolytic action, and induces osteoporosis finally [6,7].

Furthermore, excessive urinary Ca excretion can cause a decrease in the amount of antidiuretic hormone secreted at night and the amount of aquaporin 2 produced in the renal collecting duct, which affects urinary concentration [8]. Hypercalciuria can also induce changes in such renal microenvironments and cause LUTS that directly affect the patient’s quality of life, such as nocturia and nocturnal polyuria [9,10,11].

Hence, we hypothesized that such excessive urinary Ca excretion may have some effect on LUTS not only in children but also in adults. However, no clinical studies have investigated the relationship between urinary Ca excretion and LUTS in adults. The purpose of this study was to clarify the relationship between LUTS such as OAB and nocturia, and urinary Ca excretion.

## 2. Results

### 2.1. Patients’ Characteristics

Table 1 shows the patients’ characteristics. The mean age of participants was 63.9 ± 14.8 years, while the mean body mass index (BMI) was 22.4 ± 10.1 kg/m^2^. The mean urinary Ca excretion volume, measured as the urinary calcium/creatinine (Ca/Cr) ratio, was 0.16 ± 0.12 Ca/Cr, while the mean estimated daily salt intake volume was 8.9 ± 2.5 g/day. A total of 133 patients (26.0%) were diagnosed with hypercalciuria. Comparing the non-hypercalciuria group (*n* = 379, 74.0%) and the hypercalciuria group, the ratio of women (*p* = 0.006), mean age (*p* < 0.001), estimated daily salt intake volume (*p* < 0.001), and prevalence of hypertension (*p* = 0.011) in the hypercalciuria group were significantly higher than those in the non-hypercalciuria group. By contrast, the BMI of the non-hypercalciuria group was significantly higher than that of the hypercalciuria group (*p* = 0.016). The total number of cases that met the diagnostic criteria for overactive bladder (OAB) was 139 (27.2%), with 80 patients (21.1%) in the non-hypercalciuria group and 59 patients (44.4%) in the hypercalciuria group (*p* < 0.001).

### 2.2. Relationship between Urinary Calcium Excretion and LUTS

Table 2 shows the differences in the LUTS between the two groups. All items of questions and total scores for the overactive bladder symptom score (OABSS) were higher in the hypercalciuria group than in the non-hypercalciuria group (all *p* < 0.001). In the International Prostate Symptom Score (IPSS), the Q2 (frequency), Q4 (urgency), Q7 (nighttime frequency), and total scores were significantly higher in the hypercalciuria group than in the non-hypercalciuria group (all *p* < 0.001). In the subscale score comparison between the two groups, storage symptoms (Q2 + Q4 + Q7) were significantly higher in the hypercalciuria group than in the non-hypercalciuria group (*p* < 0.001); however, in voiding symptoms (Q1 [incomplete emptying] + Q3 [intermittency] + Q5 [weak stream] + Q6 [straining]), there was no statistically significant difference between the two groups (*p* = 0.722). In addition, there was no obvious difference in the IPSS-QOL scores between the two groups (*p* = 0.638).

### 2.3. Relationship between Urinary Calcium and Frequency Volume Chart

In the 3-day frequency volume chart (FVC), compared with the non-hypercalciuria group, the hypercalciuria group had significantly higher daytime frequency, nighttime frequency, proportion of the patients with nocturia (≥2), 24-h urine volume, nocturnal urine volume, nocturnal polyuria index (NPi), and ratio of patients with nocturnal polyuria (all *p* < 0.001). In addition, the voided volume in the hypercalciuria group was smaller than that in the non-hypercalciuria group (*p* < 0.001). However, there was no significant difference in diurnal urine volume between the two groups (*p* = 0.460) (Table 2).

### 2.4. Relationship between Urinary Calcium Excretion and Severity of LUTS

Through correlation analyses, the urinary Ca excretion volume was positively correlated with all questions of the OABSS (Q1, *r* = 0.232 and *p* < 0.001; Q2, *r* = 0.317 and *p* < 0.001; Q3, *r* = 0.302 and *p* < 0.001; Q4, *r* = 0.193 and *p* < 0.001) and total score (*r* = 0.347, *p* < 0.001). In addition, the urinary Ca excretion volume was positively correlated with daytime frequency (*r* = 0.144, *p* = 0.001), nighttime frequency (*r* = 0.324, *p* < 0.001), 24-h urine volume (*r* = 0.185, *p* < 0.001), and nocturnal urine volume (*r* = 0.399, *p* < 0.001), and NPi (*r* = 0.407, *p* < 0.001). By contrast, voided volume was negatively associated with urinary Ca excretion (*r* = −0.180, *p* < 0.001) (Table 3).

### 2.5. Predictive Marker for OAB Using Univariate and Multivariate Analysis

Based on these results, we performed multivariate analyses to clarify the independent effects of urinary Ca excretion on OAB, nocturia, and nocturnal polyuria (Table 4). Univariate analyses showed that hypercalciuria, female sex, age, hypertension, diabetes mellitus, renal dysfunction, and osteoporosis were significantly associated with the prevalence of OAB. Multivariate analyses showed that hypercalciuria was an independent risk factor for OAB in addition to sex, age, hypertension, and renal dysfunction (Table 4). Regarding nocturia, excessive salt intake, hypercalciuria, hypertension, and diabetes mellitus were independent risk factors in multivariate analyses. Age, salt intake volume, and hypercalciuria were found to be independent risk factors for nocturnal polyuria (Table 4).

### 2.6. Relationship between Urinary Calcium Excretion and Lower Urinary Symptoms Based on Propensity Score Matching

Table 5 shows the baseline characteristics using propensity score matching. The study analyzed the data of 200 patients (100 patients in each group cohort). The standardized mean difference of all characteristics was < 0.1, indicating negligible baseline differences between the groups. The incidence of OAB (*p* = 0.001; 95% confidence interval [CI], 1.53–5.58; odds ratio [OR], 2.92), nocturia (*p* = 0.046; 95% CI, 1.10–1.62; OR, 1.32), and nocturnal polyuria (*p* = 0.046; 95% CI, 1.05–3.24; OR, 1.84) was significantly higher in the hypercalciuria group than in the non-hypercalciuria group (Table 6).

### 2.7. Relationship between the Presence Rate of Various Urinary Parameters and Salt Intake and Urinary Calcium Excretion

**Figure 1:**
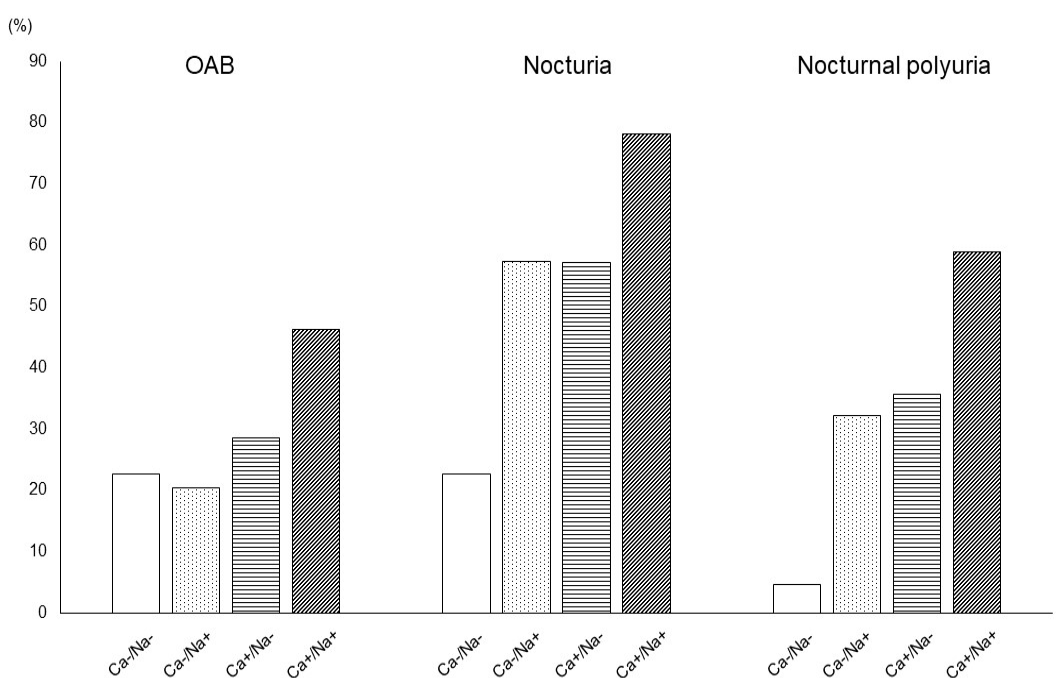
Relationship between the presence rate of various urinary parameters and salt intake and urinary calcium excretion.

Based on the results of our multivariate analysis, we examined the correlation between salt intake and urinary calcium excretion and various micturition parameters (OAB, nocturia, nocturnal polyuria) in further detail (Figure 1). Patients with excessive salt intake and hypercalciuria also had a higher incidence of all three analyzed urological parameters.

Ca−, negative for excessive calcium excretion; Ca+, positive for excessive calcium excretion; Na-, negative for excessive salt intake; Na+, positive for excessive salt intake. The presence rates of OAB (Ca−/Na−, 22.7%; Ca−/Na+, 20.3%; Ca+/Na−, 28.6%; Ca+/Na+, 46.2%; *p* < 0.001), nocturia (Ca−/Na−, 22.7%; Ca−/Na+, 57.4%; Ca+/Na−, 57.1%; Ca+/Na+, 78.2%; *p* < 0.001), and nocturnal polyuria (Ca−/Na−, 4.7%; Ca−/Na+, 32.3%; Ca+/Na−, 35.7%; Ca+/Na+, 58.8%; *p* < 0.001) of the Ca+/Na+ group were higher than those of any other group.

## 3. Discussion

The present study revealed that hypercalciuria was associated with LUTS, especially storage symptoms, including daytime frequency, nocturia, urgency, and decreased voided volume. In addition, hypercalciuria was associated with nighttime urine production. Furthermore, univariate and multivariate analyses showed that hypercalciuria was an independent risk factor for the prevalence of OAB, nocturia, and increased nocturnal polyuria.

In this study, urinary Ca excretion was calculated using the first morning urine samples with reference to previous studies, and a urinary Ca/Cr of ≥0.21 was defined as hypercalciuria [12].

Normally, the evaluation of urinary Ca excretion using 24-h urine collection to distinguish the presence of hypercalciuria is the most appropriate method. However, in the case of this study, which mainly targets outpatients, it is extremely difficult for patients to collect urine at home for 24 h. Previous studies have confirmed that the diagnosis of hypercalciuria is valid to some extent, with both sensitivity and specificity exceeding 90%, even if spot urine samples are used, including first morning samples [13,14,15].

In this study, 133 patients (26.0%) met the diagnostic criteria for hypercalciuria. There are no large epidemiological studies on the prevalence of hypercalciuria in adults. While it was reported that the prevalence of hypercalciuria was as low as 0.6% in Japanese children [16], other researchers reported that the prevalence of hypercalciuria is relatively high at 3.8–47.7% in children who complain of LUTS, and that age and region are important factors in the development of hypercalciuria [17,18]. Although a detailed examination is required, the results of this study targeting patients with some LUTS are considered to reflect the actual situation of hypercalciuria in adult patients with LUTS.

In the present study, urinary Ca excretion did not affect subjective voiding symptoms. However, all OABSS items, total score, and storage symptoms of IPSS in the hypercalciuria group were significantly higher than those in the normal group. Furthermore, in the hypercalciuria group, the daytime and nighttime frequencies were higher, and the voided volume was lower than that in the normal group. In the field of pediatric urology in particular, the relationship between hypercalciuria and OAB, such as urinary urgency, has been attracting attention. Although the detailed mechanism of the onset of OAB due to hypercalciuria is unclear, a recent report indicated that when hypercalciuria and hyperoxaluria co-exist, fine prenucleation nanoclusters of about 1 nm are formed at first, and the crystals gradually grow, depending on the conditions [19]. In addition, Rossi et al. suggested that the ions/molecules contained in drinking water play an important role in the formation and growth of calcium crystals [20]. In other words, oligomineral water rich in magnesium (Mg) and sulfate reinforces the structure of water. They demonstrated its increase of interfacial energy and induction of the formation of large numbers of small crystals. As a result, over time, some crystals, especially calcium crystals, are oriented towards growing, leading to the formation of large crystals and agglomerates of particles [20]. Furthermore, chronic bladder irritation caused by these crystal components could cause urinary storage symptoms [1,21]. Additionally, in an experiment using a rat model of hypercalciuria, Akil et al. reported that hypercalciuria can cause damage to urothelial cells and fibrosis of the bladder stroma, leading to various LUTS [10].

However, excessive urinary Ca excretion also affects the secretion of antidiuretic hormones and the production of aquaporins. In another rat model of hypercalciuria, it was demonstrated that the secretion of antidiuretic hormone is reduced, and antidiuretic hormone affects a series of reactions that trigger aquaporin 2 production by binding to V2 receptors in the collecting duct of the renal cortex [11]. In other words, it is considered that the reabsorption of water in the kidney is inhibited as a result of the reduction in aquaporin production due to the decrease in the secretion of antidiuretic hormone, which may cause polyuria [11,22]. In our study, the hypercalciuria group had a higher nocturnal urine volume and a higher NPi, although there was no difference in diurnal urine volume compared to the normal group. Although we did not evaluate antidiuretic hormones in this study, antidiuretic hormones are usually secreted during nighttime sleep. Hence, the decrease in antidiuretic hormone secretion due to hypercalciuria might have increased the amount of nocturnal urine. Furthermore, we did not confirm the patient’s water intake in this study. Hence, it cannot be ruled out that patients with hypercalciuria may have consumed more water after the evening than patients with non-hypercalciuria.

Furthermore, urinary Ca excretion is coupled with sodium (Na) excretion [5]. The present study also showed that patients with hypercalciuria had higher estimated salt intakes and higher urinary Na excretions. Some researchers have previously shown that increased urinary Na excretion suppresses Ca reabsorption by Na^+^/Ca^2+^ exchangers in the proximal tubule, resulting in increased urinary Ca excretion [4,23]. Excessive salt intake is thought to increase urinary Na excretion and increase the production of nocturnal urine [24]. In addition, the results of this study have led us to speculate that the increase in urinary Na excretion indirectly increases Ca excretion and affects nocturnal urine volume. Based on these views, interventions in LUTS, such as nocturnal enuresis by diet therapy, a Ca-restricted, and Na-restricted diet, have been reported [11,25].

Urinary Ca excretion is often associated with lifestyle-related diseases. In patients with hypertension caused by excessive salt intake, urinary Ca and Na excretion increased simultaneously, as described above [4]. In chronic kidney disease, the following may cause of hypercalciuria: 1) decreased phosphorus excretion and decreased vitamin D activation and 2) increased blood Mg as a result of chronic renal dysfunction, which suppresses parathyroid hormone secretion and affects Ca channels in the distal convoluted tubules and collecting ducts [4,26,27]. Furthermore, it is often reported that lifestyle-related diseases that cause hypercalciuria are risk factors for LUTS, such as OAB and nocturia [18,28]. In the multivariate analysis in this study, the presence of hypertension was a risk factor for nocturia and OAB. Furthermore, the present study showed that renal dysfunction was associated with OAB and that diabetes was associated with nocturia. However, not all lifestyle-related diseases were associated with the prevalence of nocturnal polyuria. In particular, the lifestyle-related disease osteoporosis did not affect any of the items of OAB, nocturia, or nocturnal polyuria. In this regard, urinary Ca excretion seems to be affected by therapeutic agents for lifestyle-related diseases. For example, loop diuretics used for hypertension and renal dysfunction promote urinary Ca excretion, while thiazide diuretics suppress urinary Ca excretion [29]. In addition, it is widely known that osteoporosis therapeutic regimens, such as vitamin D agents, promote urinary Ca excretion, while some bisphosphonates suppress bone resorption and reduce urinary Ca excretion. The effect of therapeutic agents for lifestyle-related diseases on Ca excretion was not considered in this study, and further detailed studies are required in the future. However, in this study we clarified that hypercalciuria was associated with the presence of several LUTS, meaning that it is desirable to construct a treatment strategy that considers urinary Ca excretion in patients with lifestyle-related diseases with LUTS.

This study has several limitations. This was a cross-sectional study with a relatively small number of patients and no comparison with the control group without LUTS. Furthermore, urinary Ca excretion was measured using the first morning urine samples instead of 24-h urine collection samples, and serum Ca levels were not evaluated. In addition, we did not examine the cause of hypercalciuria in detail, nor the antidiuretic hormone and water intake volume of the patients. Some causes of polyuria are dietary, idiopathic, and secondary, and patients with hypercalcemia are known to decrease the secretion of antidiuretic hormone [11,30]. We speculate that such factors may cause LUTS such as OAB and nocturia. As revealed in this study, hypercalciuria was associated with an increase in urinary Na excretion and is considered to be associated with various pathological conditions. In this study, we showed for the first time that hypercalciuria can be a risk factor for OAB, nocturia, and nocturnal polyuria using propensity score matching analysis and multivariate analyses, and we consider this to be a very important finding. Hence, we speculate that hypercalciuria has a surrogate marker-like function at the onset of LUTS rather than causing LUTS directly. To clarify these problems, it is necessary to conduct a detailed study on the correlation and confounding between urinary Na excretion, salt intake, and urinary Ca excretion. Furthermore, it is necessary to conduct a prospective study to evaluate the effects of diet and medication on urinary Ca excretion and LUTS in patients with hypercalciuria by comparing them with the control group with placebo treatment. However, calcium intake varies widely from region to region. In particular, Japanese are known to have lower calcium intake than Americans and French [31,32]. In addition, several Japanese individuals do not meet the calcium intake (650–800 mg/day) criterion recommended by the National Health and Nutrition Survey of Japan [33,34]. Furthermore, chronic calcium intake deficiency is associated with the development of cerebrovascular disease and cognitive function and can also cause the impaired activities of daily living in patients [35,36,37]. Hence, recommending calcium restriction without detailed consideration of the general comorbidities of patients with LUTS should be considered. When conducting a detailed prospective study, it is necessary to set a research method that matches the cause of hypercalcemia.

## 4. Materials and Methods

### 4.1. Patients

**Figure 2:**
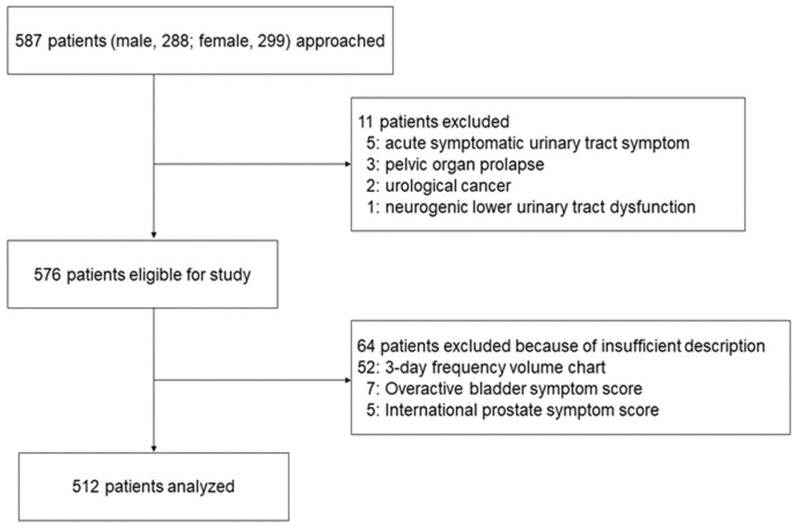
Patient flow diagram.

The present cross-sectional clinical study was carried out from October 2015 to March 2018, including patients with LUTS at Nagasaki University Hospital, Nagasaki, Japan. We conducted this study with 587 consecutive patients over 20 years of age (288 men and 299 women). Five patients with acute symptomatic urinary tract infection, three with pelvic organ prolapse, two with urological cancer, and one with neurogenic lower urinary tract dysfunction, as well as seven and five patients who missed OABSS and IPSS, respectively, were excluded in this study. Furthermore, 52 patients with insufficient descriptions of the 3-day FVC were also excluded. Finally, various parameters of 512 patients included in this study were evaluated (Figure 2).

### 4.2. Evaluation of Vital Signs

Hypertension was defined as a systolic blood pressure of ≥140 mmHg and/or a diastolic blood pressure of ≥90 mmHg or receiving therapy for hypertension. The patients’ blood pressure was evaluated using an automated oscillometric upper-arm blood pressure monitoring device (HBP-9020, Omron Co., Kyoto, Japan) in our institution. The participants were seated in a quiet room at a comfortable temperature and were instructed to avoid talking during the procedure. Blood pressure measurements were started after the participants had rested for 5 min. The participants sat on a chair with their legs uncrossed and their feet flat on the floor. All blood pressure measurements were performed on the participant’s left arm at the level of the heart. Data were measured twice at intervals of approximately 2 min, and the average value was used as the measured value. Body height (cm) and weight (kg) were recorded using a weighing machine (AD-6107NP, A&D Co., Tokyo, Japan) and stadiometer (HS20, TSUTUMI Co., Tokyo, Japan). We used the data to calculate BMI using the standard formula (BMI = weight [kg]/height [m]^2^) at outpatient visits. Diurnal urine volume was defined as the total urine volume voided from the first morning void to the last void before bed. Nocturnal urine volume was defined based on the ICS standardization: total volume of urine passed during the night, including the first morning void. NPi was defined as the ratio of nocturnal to 24-h total urine production [38]. We used a definition of two or more episodes of urination per night to identify nocturia in the univariate and multivariate analyses in this study. In addition, we defined nocturnal polyuria as present when >10 mL/kg body of urine was produced at night [39]. Renal dysfunction was defined as an estimated glomerular filtration rate of <60 mL/min/1.73 m^2^. In addition, osteoporosis was defined as having a medical history of fragile fractures or being prescribed medications under the diagnosis of osteoporosis by family doctors.

### 4.3. Evaluation of Daily Salt Intake and Urinary Calcium Volume

We used the first morning and spot urine samples to estimate urinary Ca excretion and daily salt intake volume, respectively. The urinary Ca concentration was corrected using the urinary Cr concentration (mg/dL). The group of patients with a urinary Ca/Cr value of ≥0.21 was defined as the hypercalciuria group, and the group of patients with a urinary Ca/Cr value of < 0.21 was defined as the non-hypercalciuria group [12]. We measured the urinary Ca concentration and the urinary Cr concentration with the methyl xylenol blue method and the enzymatic method, respectively, using an automatic biochemical analyzer (BioMajesty TM JCA-BM6070, JEOL Ltd., Tokyo, Japan). Daily salt intake was recorded by estimating the Na and Cr concentrations in spot urine samples, as described previously [40]. Thereafter, to estimate the Na and Cr concentrations in the spot urine samples from the daily salt intake, we used a formula that was adjusted for body height, weight, and age as follows: 24-h Na excretion (mEq/day) = 21.98 (Na S/[Cr S × 10] × Pr.UCr24)^0.392^ − 2.04 × age (years) + 14.89 × body weight (kg) + 16.14 × height (cm) − 2244.45, where Na S is the Na concentration in the spot urine sample in mEq/L, Cr S is the Cr concentration in the spot urine sample in mg/dL, and Pr.UCr24 is the predicted 24-h urinary Cr excretion in mg/day. This formula appeared to be less affected by the sample collection time, and its accuracy was relatively high. In addition, we defined excessive salt intake as 8 g/day or more for men and 7 g/day for women [41].

### 4.4. Evaluation of LUTS

We examined the relationship between LUTS (evaluated using the OABSS and IPSS) [42,43] and urinary Ca excretion. In particular, the participants were divided into two groups, the hypercalciuria group and the non-hypercalciuria group, depending on the urine examination described above. We evaluated the differences in subjective symptoms between the two groups. We defined the OAB according to the OABSS (OABSS ≥ 2 for urgency and a total OABSS  ≥ 3) [43], and the differences in each parameter between the groups were compared and examined in detail. In addition, we asked the participants to complete a 3-day FVC and evaluated the average urine volume, voided volume, and frequency of the 3-day data by the presence or absence of hypercalciuria. Furthermore, univariate and multivariate analyses were used to assess whether hypercalciuria could be a risk factor for OAB, nocturia, and nocturnal polyuria.

### 4.5. Propensity Score Matching

Patients with hypercalciuria were matched (1:1 ratio) with those who were non-hypercalciuria according to their propensity score through nearest neighbor matching based on their characteristics. We set the caliper width of 0.2 SDs.

### 4.6. Statistical Analysis

All data and values are expressed as mean ± SD, and Student’s t-test or Mann–Whitney U test was performed as needed. The χ^2^-test was used for categorical comparisons. Pearson’s correlation and the correlation coefficient (*r*) were used to evaluate the relationship between continuous variables, and the corresponding *p*-values are shown. Crude and adjusted effects were estimated by logistic regression analysis and described as OR with 95% CIs, along with *p*-values. All statistical tests were performed using JMP 15 (SAS Institute, Cary, NC, USA). The number of samples was determined on the basis of previous reports [17,18,44]. Hence, we set a probability of 0.05 (two-sided), a power of 80%, and an effect size of 0.5. We estimated that the ideal number of participants for this study should be at least 462.

## 5. Conclusions

This is the first report to show that hypercalciuria is associated with OAB, nocturia, and nocturia in adults. In the future, it will be necessary to consider treatment options for patients with LUTS and hypercalciuria.

## Figures and Tables

**Table 1:** Patients’ characteristics by the presence or absence of hypercalciuria.

	Entire *n* = 512	Non-Hypercalciuria *n* = 379	Hypercalciuria *n* = 133	*p*-Value
Gender (male/female)	241/271	192/187	49/84	0.006
Age (years)	63.9 ± 14.8	62.0 ± 15.3	69.5 ± 11.6	<0.001
Body mass index (Kg/m^2^)	22.4 ± 10.1	22.9 ± 11.1	21.0 ± 6.4	0.016
Urinary calcium excretion volume (urinary Ca/Cr)	0.16 ± 0.12	0.10 ± 0.05	0.32 ± 0.11	<0.001
Estimated daily salt intake volume (g/day)	8.9 ± 2.5	8.4 ± 2.2	10.2 ± 2.7	<0.001
Overactive bladder (%)	139 (27.2)	80 (21.1)	59 (44.4)	<0.001
Hypertension (%)	180 (35.2)	121 (31.9)	59 (44.4)	0.011
Diabetes mellitus (%)	47 (9.2)	40 (10.6)	7 (5.3)	0.081
Renal dysfunction (%)	82 (16.0)	60 (15.8)	22 (16.5)	0.891
Hyperlipidemia (%)	58 (11.3)	42 (11.1)	16 (12.0)	0.752
Osteoporosis (%)	47 (9.2)	34 (9.0)	13 (9.2)	0.862

**Table 2:** Urological condition of participants by the presence or absence of hypercalciuria.

	Entire *n* = 512	Non-Hypercalciuria *n* = 379	Hypercalciuria *n* = 133	*p*-Value
OABSS				
Q1 (daytime frequency)	0.5 ± 0.6	0.4 ± 0.6	0.7 ± 0.7	<0.001
Q2 (nighttime frequency)	1.5 ± 1.0	1.3 ± 1.0	2.1 ± 0.8	<0.001
Q3 (urgency)	0.9 ± 1.2	0.7 ± 1.2	1.5 ± 1.2	<0.001
Q4 (urgency incontinence)	0.3 ± 0.9	0.3 ± 0.8	0.5 ± 0.9	<0.001
Total score	3.2 ± 2.9	2.7 ± 2.8	4.7 ± 2.8	<0.001
IPSS				
Q1 (incomplete emptying)	1.4 ± 1.5	1.4 ± 1.6	1.3 ± 1.1	0.901
Q2 (frequency)	1.0 ± 1.2	0.8 ± 1.0	1.4 ± 1.5	<0.001
Q3 (intermittency)	0.9 ± 1.2	0.9 ± 1.2	0.9 ± 1.3	0.834
Q4 (urgency)	0.9 ± 1.0	0.8 ± 1.0	1.2 ± 1.1	<0.001
Q5 (weak stream)	1.7 ± 1.3	1.7 ± 1.3	1.6 ± 1.1	0.684
Q6 (straining)	1.3 ± 1.5	1.3 ± 1.5	1.4 ± 1.4	0.195
Q7 (nighttime frequency)	1.7 ± 1.1	1.5 ± 1.1	2.2 ± 1.2	<0.001
Storage symptoms (Q2 + Q4 + Q7)	3.6 ± 2.4	3.1 ± 2.1	4.8 ± 2.6	<0.001
Voiding symptoms (Q1 + Q3 + Q5 + Q6)	5.3 ± 3.7	5.3 ± 3.8	5.2 ± 3.2	0.722
Total score	8.8 ± 4.7	8.4 ± 4.7	10.0 ± 4.5	<0.001
QOL score	3.7 ± 1.4	3.7 ± 1.4	3.7 ± 1.2	0.638
Frequency volume chart				
Number of daytime frequency	7.5 ± 2.3	7.3 ± 2.3	8.0 ± 2.2	<0.001
Number of nighttime frequency	1.7 ± 1.3	1.4 ± 1.2	2.5 ± 1.3	<0.001
Nocturia ≥2 (%)	274 (53.5)	173 (45.6)	101 (75.9)	<0.001
24-h urine volume (mL)	2136.0 ± 561.5	2086.2 ± 568.2	2278.0 ± 518.1	<0.001
Diurnal urine volume (mL)	1662.8 ± 437.6	1658.2 ± 450.4	1675.9 ± 400.1	0.460
Nocturnal urine volume (mL)	473.2 ± 230.5	428.0 ± 209.2	602.0 ± 240.4	<0.001
Nocturnal polyuria index (%)	21.7 ± 8.0	20.1 ± 7.3	26.2 ± 8.1	<0.001
Presence of noctornal polyuria (%)	162 (31.6)	87 (23.0)	75 (56.4)	<0.001
Voided volume (mL)	240.5 ± 38.3	246.7 ± 37.5	222.7 ± 34.8	<0.001

OABSS, overactive bladder symptom score; IPSS, international prostate symptom score.

**Table 3:** Relationship between urinary calcium excretion volume and the severity of OABSS.

	*r*	*p*-Value
OABSS		
Q1 (daytime frequency)	0.232	<0.001
Q2 (nighttime frequency)	0.317	<0.001
Q3 (urgency)	0.302	<0.001
Q4 (urgency incontinence)	0.193	<0.001
Total score	0.347	<0.001
Number of daytime frequency	0.144	0.001
Number of nighttime frequency	0.324	<0.001
24-h urine volume (mL)	0.185	<0.001
Diurnal urine volume (mL)	0.028	0.533
Nocturnal urine volume (mL)	0.399	<0.001
Nocturnal polyuria index (%)	0.407	<0.001
Voided volume (mL)	−0.180	<0.001

OABSS, overactive bladder symptom score.

**Table 4:** Overactive bladder and urinary symptom-related factors.

	Univariate Analysis				Multivariate Analysis		
	OR	95% CI	*p* Value		OR	95% CI	*p* Value
For overactive bladder							
Gender: male	0.51	0.34–0.76	<0.001		0.55	0.35–0.86	0.009
Age	1.04	1.02–1.05	<0.001		1.02	1.01–1.03	0.004
Body mass index	1.00	0.98–1.02	0.656		-	-	-
Estimated daily salt intake volume	1.03	0.96–1.12	0.387		-	-	-
Hypercalciuria: presence	2.98	1.95–4.55	<0.001		2.61	1.66–4.11	<0.001
Hypertension: presence	1.89	1.27–2.82	0.002		1.57	1.44–1.67	0.032
Diabetes mellitus: presence	1.95	1.04–3.60	0.038		1.82	0.28–1.08	0.082
Renal dysfunction: presence	2.48	1.52–4.05	<0.001		2.04	1.17–3.54	0.014
Hyperlipidemia: presence	1.24	0.67–2.21	0.480		-	-	-
Osteoporosis: presence	2.15	1.15–3.97	0.017		1.42	0.71–2.83	0.324
For nocturia (≥2)							
Gender: male	0.75	0.50–1.13	0.171		-	-	-
Age	1.00	0.99–1.03	0.965		-	-	-
Body mass index	0.99	0.98–1.02	0.630		-	-	-
Estimated daily salt intake volume	1.48	1.34–1.65	<0.001		1.48	1.33–1.66	<0.001
Hypercalciuria: presence	3.87	2.52–5.97	<0.001		2.16	1.33–3.5	0.002
Hypertension: presence	1.99	1.32–2.99	<0.001		2.91	1.81–4.73	<0.001
Diabetes mellitus: presence	5.45	1.94–22.8	<0.001		7.00	2.23–31.5	<0.001
Renal dysfunction: presence	1.05	0.62–1.86	0.854		-	-	-
Hyperlipidemia: presence	1.50	0.78–3.13	0.248		-	-	-
Osteoporosis: presence	2.03	0.94–5.07	0.094		-	-	-
For nocturnal polyuria (>10 mL/kg body)							
Gender: male	0.83	0.57–1.28	0.336		-	-	-
Age	1.03	1.01–1.05	<0.001		1.02	1.01–1.06	<0.001
Body mass index	1.01	0.99–1.05	0.292		-	-	-
Estimated daily salt intake volume	1.47	1.34–1.63	<0.001		1.41	1.28–1.58	<0.001
Hypercalciuria: presence	4.34	2.87–6.62	<0.001		2.73	1.69–441	<0.001
Hypertension: presence	1.37	0.93–2.01	0.111		-	-	-
Diabetes mellitus: presence	2.41	1.16–5.68	0.017		1.87	0.82–4.78	0.141
Renal dysfunction: presence	1.07	0.65–1.80	0.806		-	-	-
Hyperlipidemia: presence	2.51	1.11–4.43	0.022		1.89	0.92–4.19	0.085
Osteoporosis: presence	1.38	0.73–2.55	0.310		-	-	-

OR, odds ratio; CI, confidence interval.

**Table 5:** Baseline characteristic data of hypercalciuria after propensity score matching.

	Non-Hypercalciuria *n* = 100	Hypercalciuria *n* = 100	*p* Value	Standardized Mean Differences
Gender (male/female)	36/64	43/57	0.386	0.085
Age (years)	68.2 ± 12.6	68.6 ± 12.6	0.801	0.036
Body mass index (Kg/m^2^)	21.6 ± 5.2	21.6 ± 5.2	0.958	0.015
Estimated daily salt intake volume (g/day)	9.5 ± 1.9	9.5 ± 2.0	0.951	0.009
Hypertension (%)	43 (43.0)	42 (42.0)	1.000	0.020
Diabetes mellitus (%)	9 (9.0)	7 (7.0)	0.795	0.074
Renal dysfunction (%)	20 (20.0)	16 (16.0)	0.581	0.094
Hyperlipidemia (%)	15 (15.0)	13 (13.0)	0.839	0.058
Osteoporosis (%)	14 (14.0)	9 (9.0)	0.376	0.008
24-h urine volume (mL)	2255.4 ± 639.0	2246 ± 520.1	0.913	0.015

**Table 6:** Differences in lower urinary tract symptoms between the presence and absence of hypercalciuria based on propensity score matching methods.

	Non-Hypercalciuria *n* = 100	Hypercalciuria *n* = 100	*p* Value
OAB (%)	19 (19.0)	42 (42.0)	0.001
Nocturia ≥2 (%)	55 (55.0)	45 (45.0)	0.028
Nocturnal polyuria >10 mL/kg body (%)	37 (37.0)	52 (52.0)	0.046

OAB, overactive bladder.

## Data Availability

The data presented in this study are available on request from the corresponding author. The data are not publicly available due to privacy/ethical restrictions.
